# Molecular Features of Resected Melanoma Brain Metastases, Clinical Outcomes, and Responses to Immunotherapy

**DOI:** 10.1001/jamanetworkopen.2023.29186

**Published:** 2023-08-17

**Authors:** Harish N. Vasudevan, Cyrille Delley, William C. Chen, Kanish Mirchia, Sixuan Pan, Poojan Shukla, Alex A. Aabedi, Minh P. Nguyen, Ramin A. Morshed, Jacob S. Young, Lauren Boreta, Shannon E. Fogh, Jean L. Nakamura, Philip V. Theodosopoulos, Joanna Phillips, Shawn L. Hervey-Jumper, Mariza Daras, Luke Pike, Manish K. Aghi, Katy Tsai, David R. Raleigh, Steve E. Braunstein, Adam R. Abate

**Affiliations:** 1 Department of Radiation Oncology, University of California, San Francisco; 2 Department of Neurological Surgery, University of California, San Francisco; 3 Department of Bioengineering, University of California, San Francisco; 4 Department of Pathology, University of California, San Francisco; 5 Department of Radiation Oncology, Memorial Sloan Kettering Cancer Center, New York, New York; 6 Department of Hematology/Oncology, University of California, San Francisco

## Abstract

**Importance:**

Central nervous system (CNS)–penetrant systemic therapies have significantly advanced care for patients with melanoma brain metastases. However, improved understanding of the molecular landscape and microenvironment of these lesions is needed to both optimize patient selection and advance treatment approaches.

**Objective:**

To evaluate how bulk and single-cell genomic features of melanoma brain metastases are associated with clinical outcome and treatment response.

**Design, Setting, and Participants:**

This cohort study analyzed bulk DNA sequencing and single nuclear RNA-sequencing data from resected melanoma brain metastases and included 94 consecutive patients with a histopathologically confirmed diagnosis of melanoma brain metastasis who underwent surgical resection at a single National Comprehensive Cancer Network cancer center in San Francisco, California, from January 1, 2009, to December 31, 2022.

**Exposure:**

A Clinical Laboratory Improvement Amendments–certified targeted sequencing assay was used to analyze tumor resection specimens, with a focus on *BRAF* V600E alteration. For frozen pathologic specimens from CNS treatment-naive patients undergoing surgical resection, commercial single nuclear RNA sequencing approaches were used.

**Main Outcomes and Measures:**

The primary outcome was overall survival (OS). Secondary outcomes included CNS progression-free survival (PFS), microenvironmental composition with decreased T-cell and macrophage populations, and responses to immunotherapy.

**Results:**

To correlate molecular status with clinical outcome, Kaplan-Meier survival analysis of 94 consecutive patients (median age, 64 years [range, 24-82 years]; 70 men [74%]) with targeted *BRAF* alteration testing showed worse median intracranial PFS (*BRAF* variant: 3.6 months [IQR, 0.1-30.6 months]; *BRAF* wildtype: 11.0 months [IQR, 0.8-81.5 months]; *P* < .001) and OS (*BRAF* variant: 9.8 months [IQR, 2.5-69.4 months]; *BRAF* wildtype: 23.2 months [IQR, 1.1-102.5 months]; *P* = .005; log-rank test) in *BRAF* V600E variant tumors. Multivariable Cox proportional hazards regression analysis revealed that *BRAF* V600E status was an independent variable significantly associated with both PFS (hazard ratio [HR], 2.65; 95% CI, 1.54-4.57; *P* < .001) and OS (HR, 1.96; 95% CI, 1.08-3.55; *P* = .03). For the 45 patients with resected melanoma brain metastases undergoing targeted DNA sequencing, molecular classification recapitulated The Cancer Genome Atlas groups (*NRAS* variant, *BRAF* variant, *NF1* variant, and triple wildtype) with no subtype enrichment within the brain metastasis cohort. On a molecular level, *BRAF* V600E variant lesions were found to have a significantly decreased tumor mutation burden. Moreover, single nuclear RNA sequencing of treatment-naive *BRAF* V600E variant (n = 3) brain metastases compared with *BRAF* wildtype (n = 3) brain metastases revealed increased immune cell populations in *BRAF* wildtype tumors (mean [SD], 11% [4.1%] vs 3% [1.6%] CD45-positive cells; *P* = .04). Survival analysis of postoperative immunotherapy responses by *BRAF* status revealed that *BRAF* wildtype lesions were associated with a response to checkpoint inhibition (median OS: with immunotherapy, undefined; without immunotherapy, 13.0 months [range, 1.1-61.7 months]; *P* = .001; log-rank test) while *BRAF* variant lesions (median OS: with immunotherapy, 9.8 months [range, 2.9-39.8 months]; without immunotherapy, 9.5 months [range, 2.5-67.2 months]; *P* = .81; log-rank test) were not.

**Conclusions and Relevance:**

This molecular analysis of patients with resected melanoma brain metastases found that *BRAF* V600E alteration is an important translational biomarker associated with worse clinical outcomes, differential microenvironmental composition, and benefit from immunotherapy. Patients with *BRAF* V600E variant melanoma brain metastases may thus benefit from alternative CNS-penetrant systemic regimens.

## Key Points

**Question:**

What is the translational importance of alteration status in the treatment of melanoma brain metastases?

**Findings:**

In this cohort study of 94 patients with resected melanoma brain metastases that underwent targeted DNA sequencing, *BRAF* V600E variant lesions were associated with worse intracranial progression-free survival and overall survival. Single-cell sequencing showed that *BRAF* V600E variant brain metastases harbored fewer immune cell types; immunotherapy was associated with improved outcomes for patients with *BRAF* wildtype but not *BRAF* V600E variant brain metastases.

**Meaning:**

This study suggests that, for patients with melanoma brain metastases, *BRAF* status may be an important molecular biomarker to guide systemic therapy selection.

## Teaser

This cohort study evaluates how bulk and single-cell genomic features of melanoma brain metastases are associated with clinical outcome and treatment response.

## Introduction

Brain metastases are a significant source of mortality among patients with metastatic melanoma, and central nervous system (CNS)–penetrant systemic regimens such as immune checkpoint inhibition (ICI) have demonstrated significant clinical responses.^1,2,3^ However, the molecular landscape of melanoma brain metastases remains poorly understood; moreover, the association between molecular characteristics and systemic therapy responses for melanoma brain metastases remains unclear. Given recent results demonstrating the superiority of ICI compared with targeted therapy for *BRAF* (OMIM 164757) variant extracranial metastatic melanoma,^4^ we performed alteration analysis and single nuclear RNA sequencing (snRNA-seq) of resected melanoma brain metastases to connect molecular features with clinical outcomes, specifically to evaluate whether intracranial metastases harbor a distinct alteration profile compared with extracranial melanoma, identify molecular alterations associated with overall survival (OS) and progression-free survival (PFS), and investigate if unique cellular subpopulations and transcriptional signatures exist between different melanoma brain metastases based on *BRAF* alteration status.

## Methods

### Patient Cohort and Tumor Characteristics

A total of 94 patients who underwent craniotomy and resection for pathologically confirmed melanoma brain metastases or biopsy in the setting of concern for leptomeningeal disease between January 1, 2009, and December 31, 2022, at a single institution with molecular assessment of *BRAF* alteration status were retrospectively identified. Using the University of California, San Francisco (UCSF) tumor registry and medical record, baseline demographic and clinical outcome data were extracted. Exclusion criteria included pathologic review identifying a primary CNS malignant neoplasm or nonmelanoma primary tumor. Patients with unresected brain metastases beyond the index lesion were included in this study without an upper limit on maximum number of metastases. Detailed cohort characteristics are provided in the Table and separated based on *BRAF* alteration status in eTable 1 in Supplement 1. For the 45 patients who underwent targeted DNA sequencing using a Clinical Laboratory Improvement Amendments (CLIA)–certified genetic testing assay spanning over 500 cancer-associated genes as part of routine clinical care, variant information was obtained from the medical record and manually reviewed by the study team to include pathogenic or likely pathogenic alterations for further analysis. For the remaining patients, the *BRAF* exon 15 alteration status was assessed by polymerase chain reaction as a part of routine clinical care; accordingly, all patients with resected, pathologically confirmed melanoma brain metastases within the study period had targeted *BRAF* sequencing performed. A full list of variants observed in the cohort is provided in eTable 2 in Supplement 1. The University of California San Francisco institutional review board approved this study. Written consent for molecular analysis of tumors was obtained as part of routine clinical care.

**Table.** Baseline Clinical Characteristics of Patients With Resected Melanoma Brain Metastases

Characteristic	Patients, No. (%) (N = 94)
Age, median (range), y	64 (24-82)
KPS, median (range)	80 (40-100)
Extracranial disease	57 (61)
Targeted DNA sequencing panel, No.	45
Extent of resection	
Gross total resection	72 (77)
Subtotal resection	15 (16)
Not assessed	7 (7)
No. of metastases	
1	26 (28)
2-5	28 (30)
6-10	9 (10)
>10	30 (32)
Postoperative radiotherapy	85 (90)
Postoperative systemic therapy	62 (66)
Postoperative immunotherapy	48 (51)
Postoperative targeted therapy	30 (32)
Multiple systemic regimens	16 (17)
Data not available	5 (5)
Preoperative radiotherapy	10 (11)
Preoperative systemic therapy	15 (16)
Preoperative immunotherapy	13 (14)
Preoperative targeted therapy	2 (2)
Multiple systemic regimens	0
Data not available	5 (5)
Follow-up, median (range) mo	12.7 (0.1-69.1)

Abbreviation: KPS, Karnofsky performance score.

### Outcomes

The primary outcome was OS. Secondary outcomes associated with *BRAF* V600E alteration include worse CNS PFS, altered microenvironmental composition with decreased T-cell and macrophage populations, and poorer responses to immunotherapy. Patients were identified from an institutional registry, and all cases were subsequently reviewed by a multidisciplinary team of neurosurgeons, neuro-oncologists, neuropathologists, neuroradiologists, and radiation oncologists to evaluate OS based on vital status in clinical documentation. Central nervous system PFS was defined as local failure at the resection cavity or emergence of a new metastasis in the CNS, including leptomeningeal disease on results of radiographic evaluation.

### Single Nuclear RNA Sequencing

A total of 6 resected, flash-frozen melanoma brain metastasis specimens with sufficient tissue for snRNA-seq were processed for single nuclear isolation. Samples were selected from treatment-naive patients comprising *BRAF* wildtype (n = 3) and *BRAF* variant (n = 3) brain metastases to test the hypothesis that distinct tumor and/or nontumor cellular populations and transcriptional programs underlie clinical differences between molecular melanoma brain metastasis groups. Specimens were thawed on ice, minced with sterile razor blades, and mechanically dounced on ice in cell lysis nuclei extraction buffer until all macroscopically visible tissue dissolved into suspension. Cell suspensions were filtered through a 50-μm filter, centrifuged at 500g for 5 minutes at 4 °C, and resuspended in 0.1% bovine serum albumin in phosphate-buffered saline. Nuclei were stained using DAPI (4′,6-diamidino-2-phenylindole; No. D3571, Thermo Fisher Scientific) and counted. A total of 10 000 nuclei were loaded per single-nuclei RNA sequencing sample. Single-nuclei sequencing was performed using the Chromium Single Cell 3′ Library & Gel Bead Kit v3.1 on a 10x Chromium controller (10X Genomics) using the manufacturer-recommended default protocol and settings. Samples were sequenced on an Illumina NovaSeq at the UCSF Center for Advanced Technology, and the resulting FASTQ files were processed using the CellRanger analysis suite^5^ for alignment to the hg38 reference genome, identification of empty droplets, and determination of a count threshold. All downstream analyses were performed in Seurat^6^ using the default pipeline. In brief, data were empirically filtered on a per-sample basis to remove outliers with regard to gene count, unique molecular identifier count, or mitochondrial genes, followed by cluster identification, Uniform Manifold Approximation and Projection (UMAP) generation, and marker gene list generation using computed highly variable features and the top ten principal component dimensions as previously described.^7^

### Statistical Analysis

Evaluation of categorical variables such as ploidy (euploid vs aneuploid), Karnofsky performance score (KPS) (<70 vs 80 vs 90-100), or extracranial disease status (present vs absent) was performed with either the χ^2^ or Fisher exact test, while unpaired *t* tests were used for comparison of the continuous variables such as age, number of brain metastases, tumor mutation burden, and microsatellite instability within our targeted sequencing clinical cohort under the assumption of a large, normally distributed sample (n = 94). Comparisons were carried out based on The Cancer Genome Atlas (TCGA) molecular group alteration status (*BRAF*, *NRAS* [OMIM 164790], *NF1* [OMIM 162200], or triple wildtype tumors) and further analysis focused on comparisons between *BRAF* V600E vs non-*BRAF* V600E variant melanoma brain metastases. Cell composition differences between samples in the single-cell data were analyzed using the Mann-Whitney test due to the small sample size within the *BRAF* variant and wildtype groups (3 in each). All *P* values were from 2-sided tests, and results were deemed statistically significant at *P* < .05. Central nervous system PFS and OS were estimated using the Kaplan-Meier method from the date of brain metastasis diagnosis and visualized in Prism, version 10.0.0 (GraphPad Software). Survival analysis, including the log-rank test, and Cox proportional hazards regression were computed using the survival package in R, versions 3.5.3 and 3.6.1 (R Group for Statistical Computing). Previously well-described prognostic variables comprising the widely used disease-specific graded prognostic assessment (age, KPS, presence of extracranial metastases, number of metastases, and *BRAF* V600E alteration) were selected a priori for inclusion in the multivariable model.^8^ The proportional hazards assumption was assessed via inspection of Schoenfeld residuals using the cox.zph function in the survival package in *R*.

## Results

### Targeted DNA Sequencing of Melanoma Brain Metastases and TCGA Melanoma Molecular Groups

**Figure 1.**
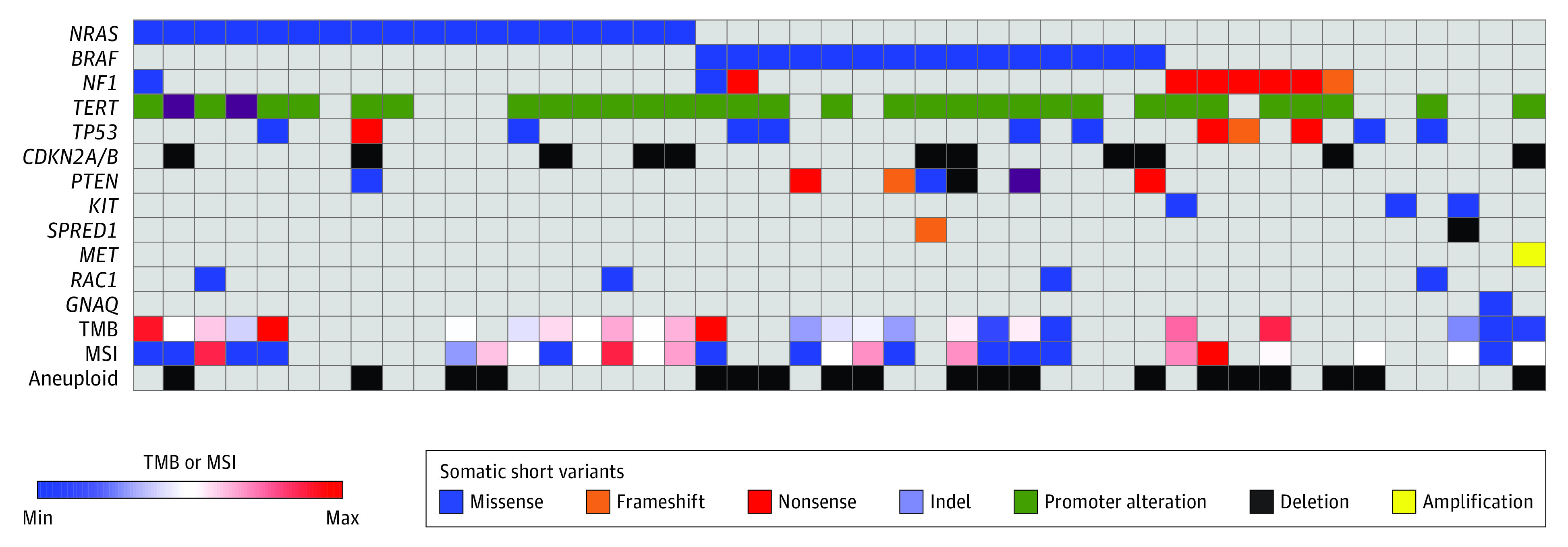
Targeted DNA Sequencing Analysis Targeted DNA sequencing analysis using a Clinical Laboratory Improvement Amendments–certified clinical genomics assay of resected melanoma brain metastases (n = 48) reveals representation across 4 consensus The Cancer Genome Atlas molecular groups and 12 recurrently mutated genes. MSI indicates microsatellite instability; and TMB, tumor mutation burden.

To test the hypothesis that melanoma brain metastases harbor a distinct alteration profile compared with extracranial melanoma, we evaluated clinical and molecular features within our cohort of 94 patients (70 men [74%] and 24 women [26%]) with resected melanoma brain metastasis and targeted *BRAF* alteration assessment (Table). The median age was 64 years (range, 24-82 years), median KPS 80 (range, 40-100), and the median follow-up was 12.7 months (range, 0.1-69.1 months). In addition, 57 patients (61%) had extracranial disease at time of diagnosis, 72 (77%) underwent gross total resection, and 26 (28%) had a single metastasis, while 30 (32%) had more than 10 metastases. Within this cohort, 45 patients were profiled using a CLIA-certified targeted next-generation sequencing panel covering more than 500 cancer-associated genes (Figure 1) (eTable 1 in Supplement 1). We first analyzed samples based on consensus TCGA melanoma molecular subgroups,^9^ confirming the presence of *NRAS* (18 of 45 [40%]), *BRAF* (15 of 45 [33%]), *NF1* (9 of 45 [20%]), and triple wildtype tumors (6 of 45 [13%]), which were mutually exclusive except for 3 cases (eFigure 1A in Supplement 2 and eTable 1 in Supplement 1). In addition, recurrent *TERT* (OMIM 187270) promoter (32 of 45 [71%]), *TP53* (OMIM 191170; 12 of 45 [27%]), *CDKN2A/B* (OMIM 600160; 11 of 45 [24%]), and *PTEN* (OMIM 601728; 7 of 45 [16%]) alterations were also observed, consistent with extracranial melanoma molecular profiling.^9^ Of the recurrent alterations defining TCGA melanoma molecular groups, only *BRAF* status is therapeutically actionable,^10^ and we thus next focused on correlations with *BRAF* alteration within our sequencing cohort. *BRAF* V600E variant tumors demonstrated a significantly decreased TAB (eFigure 1B in Supplement 2), but no difference in microsatellite instability (eFigure 1C in Supplement 2) or ploidy (eFigure 1D in Supplement 2).

### Association of *BRAF* V600E Alteration With Decreased TAB and Poor Clinical Outcomes

**Figure 2.**
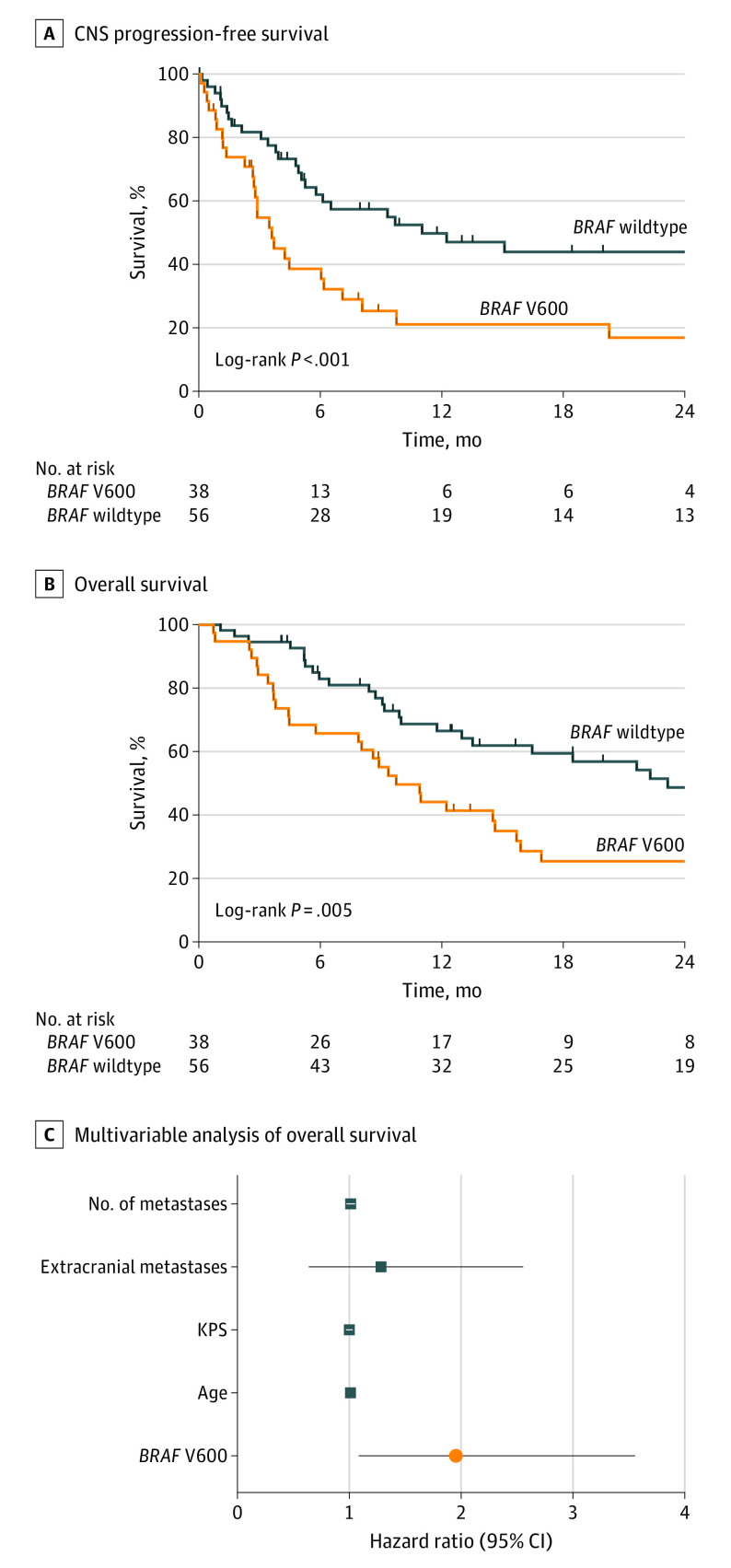
Association of *BRAF* V600E Alteration With Survival In a larger consecutive series (n = 94) of patients with resected melanoma brain metastases, *BRAF* V600E alteration was associated with significantly worse (A) central nervous system (CNS) progression-free survival and (B) overall survival. C, Multivariable analysis shows *BRAF* alteration status is significantly associated with overall survival (data plotted as mean with 95% CI). KPS indicates Karnofsky performance score.

To test the hypothesis that tumor alterations are associated with clinical outcome, we next examined the full cohort of 94 patients with resected melanoma brain metastases with targeted *BRAF* assessment. Patients with *BRAF* V600E alterations were younger (eFigure 2A in Supplement 2), but they had no significant differences in KPS (eFigure 2B in Supplement 2), extracranial disease (eFigure 2C in Supplement 2), or number of brain metastases (eFigure 2D in Supplement 2 and eTable 2 in Supplement 1). Three alternate *BRAF* V600 alterations were detected, of which 2 were *BRAF* V600K and 1 was *BRAF* V600R; these were considered in the non–*BRAF* V600E group. With regard to clinical outcome, *BRAF* V600E alteration was associated with significantly worse median CNS PFS (*BRAF* variant: 3.6 months [IQR, 0.1-30.6 months]; *BRAF* wildtype: 11.0 months [IQR, 0.8-81.5 months]; Figure 2A) and OS (*BRAF* variant: 9.8 months [IQR, 2.5-69.4 months]; *BRAF* wildtype: 23.2 months [IQR, 1.1-102.5 months]; *P* = .005; Figure 2B). When controlling for components of the melanoma brain metastases graded prognostic assessment^8^ in a multivariable Cox proportional hazards regression model, *BRAF* alteration status remained significantly associated with both OS (Figure 2C) (HR, 1.96; 95% CI, 1.08-3.55; *P* = .03) and CNS PFS (eFigure 2E in Supplement 2) (HR, 2.65; 95% CI, 1.54-4.57; *P* < .001). Taken together, these data suggest that *BRAF* alteration in melanoma brain metastases is associated with decreased TAB and worse survival outcomes.

### CD45+ Macrophage and T-Cell Subpopulations in *BRAF* V600E Variant vs Wildtype Melanoma Brain Metastases

**Figure 3.**
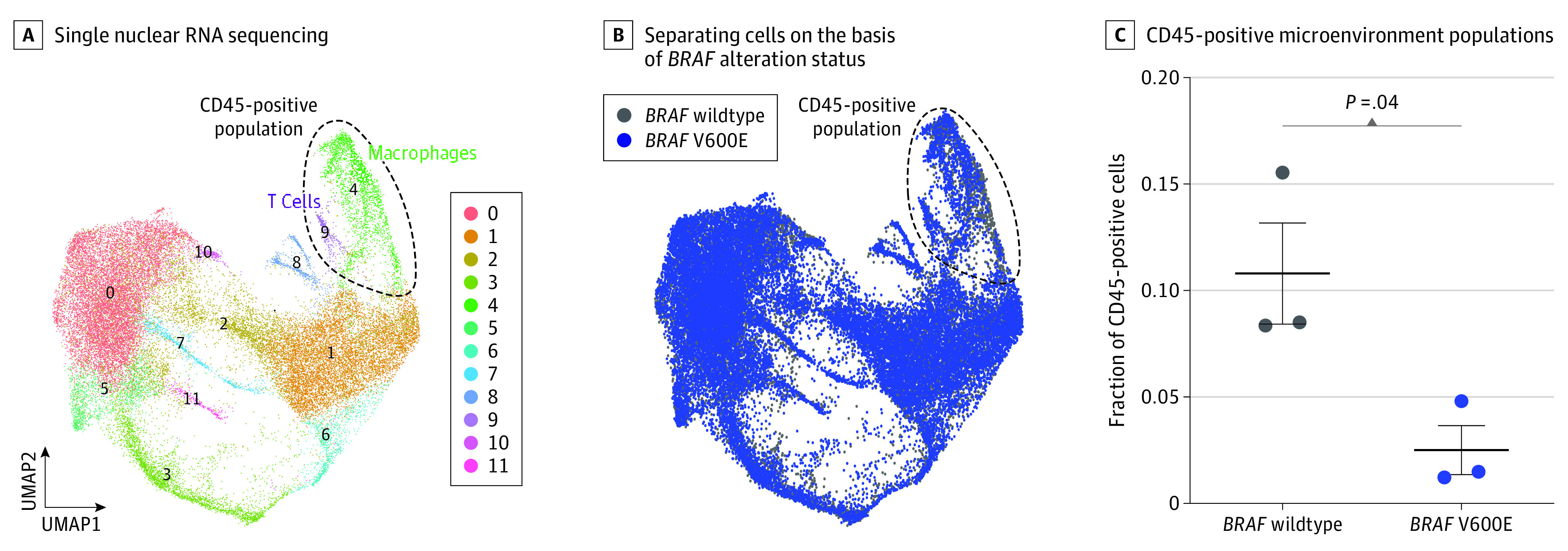
RNA Sequencing Analysis A, Single nuclear RNA sequencing of treatment-naive *BRAF* V600E variant (n = 3) and *BRAF* wildtype tumors (n = 3) reveals a total of 12 cell populations. B, Separating cells on the basis of *BRAF* alteration status demonstrates a significantly increased number of CD45-positive immune cells in *BRAF* wildtype brain metastases compared with *BRAF* V600E variant lesions. C, CD45-positive immune cells in *BRAF* wildtype brain metastases compared with *BRAF* V600E variant lesions (*P* = .04, Mann-Whitney test). UMAP indicates Uniform Manifold Approximation and Projection.

**Figure 4.**
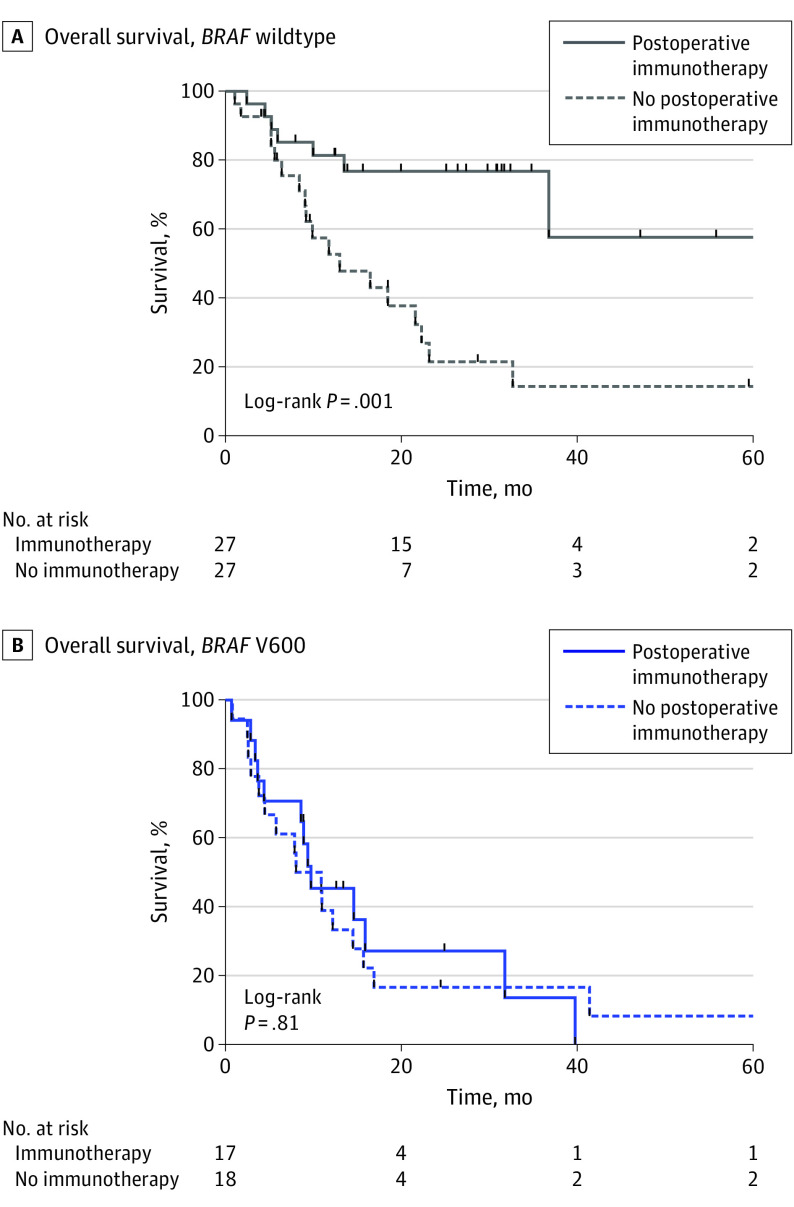
Response of Tumors to Immunotherapy A, *BRAF* wildtype tumors are associated with response to immunotherapy. B, *BRAF* V600E variant tumors did not show improved outcomes with immunotherapy.

To test the hypothesis that distinct cellular subpopulations and transcriptional programs underlie clinical differences between *BRAF* wildtype and V600E variant brain metastases, we performed snRNA-seq on treatment-naive *BRAF* wildtype (n = 3) and *BRAF* V600E variant (n = 3) melanoma brain metastases. A total of 35 527 single nuclei were isolated and analyzed, leading to the identification of 12 cellular populations by UMAP after data integration (Figure 3A; eTable 3 and eTable 4 in Supplement 1). Examination of cellular populations by *BRAF* alteration status revealed that most cellular populations were shared across multiple tumors and genotypes (clusters 0, 1, 2, 5, 6, 7, 8, and 10), while 2 of the remaining 4 clusters were enriched in *BRAF* V600E variant tumors (clusters 3 and 11) and 2 were enriched in *BRAF* wildtype tumors (clusters 4 and 9) (eFigure 3A-C in Supplement 2). Cell clusters enriched in *BRAF* V600E variant compared with *BRAF* wildtype tumors showed increased cell proliferation (eFigure 3D in Supplement 2), with a trend toward increased proportions of cluster 3 S phase cells and cluster 11 G2M phase cells (eFigure 3E in Supplement 2). Analysis of copy number variants to estimate ploidy (eFigure 3F in Supplement 2)^11^ and expression of the hematopoietic lineage marker CD45 (*PTPRC*) (eFigure 3G in Supplement 2), which was among the top cluster marker genes for clusters 4 and 9, identified cell clusters 4 and 9 as populations of nontumor CD45-positive microenvironment cells in UMAP space (Figure 3A and B) that were significantly enriched in *BRAF* wildtype compared with *BRAF* V600E variant cells (mean [SD], 11% [4.1%] vs 3% [1.6%] CD45-positive cells; *P* = .04) (Figure 3C). Further analysis of the cells in clusters 4 and 9 comprising this CD45-positive, predominantly diploid subpopulation by automated cell type classification combined with marker gene analysis revealed that cluster 4 comprised T cells marked by the pan T-cell marker CD3 (*CD3D*, *CD3E*, *CD3G*), while cluster 9 comprised macrophages marked by the macrophage marker *CD163* (eFigure 3H-I in Supplement 2). Given the significant differences in microenvironmental composition of both adaptive and innate immune cell populations based on *BRAF* status, we next examined how *BRAF* alteration status in melanoma brain metastases correlated with response to ICI immunotherapy. In our cohort, patients with *BRAF* wildtype melanoma brain metastases demonstrated significantly improved median OS with postoperative checkpoint inhibition (with immunotherapy, undefined; without immunotherapy, 13.0 months [range, 1.1-61.7 months]; *P* = .001; log-rank test) (Figure 4A), while patients with *BRAF* V600 variant melanoma brain metastases did not have improved median OS (with immunotherapy, 9.8 months [range, 2.9-39.8 months]; without immunotherapy, 9.5 months [range, 2.5-67.2 months]; *P* = .81; log-rank test) (Figure 4B). Our snRNA-seq revealed that *BRAF* V600E variant melanoma brain metastases harbored a distinct CD45-positive microenvironment compartment highlighted by decreased macrophage and T cell populations, which correlates with minimal response to checkpoint inhibition in *BRAF* V600E variant melanoma brain metastases compared with *BRAF* wildtype lesions.

## Discussion

Brain metastases are the most common intracranial tumor, yet our understanding of the molecular and cellular landscape of these lesions is only beginning to emerge.^12,13,14^ Our study is a first step in connecting bulk and single-cell genomic features to patient characteristics and outcomes to guide clinical decision-making based on molecular status. The finding that *BRAF* V600E alteration is a biomarker negatively associated with survival is contradictory to historical cohorts.^8,15^ We propose this is due, at least in part, to the poor response of *BRAF* V600E brain metastases to ICI, which has only recently become a commonly used systemic regimen for melanoma brain metastases. Moreover, our single-cell analysis showing altered microenvironments based on tumor genotype is consistent with recent melanoma brain metastasis single-cell landscaping analysis showing differential lymphocyte populations in intracranial lesions.^14^ Given that our patient cohort who underwent snRNA-seq was treatment naive, this altered microenvironment appears to be an intrinsic property of *BRAF* V600E variant melanoma brain metastases, although systemic therapies likely also play an important role in modulating the microenvironment. Our data suggest that ICI may be less efficacious in *BRAF* V600E variant melanoma brain metastases but do not address the question of whether targeted therapy (combination *BRAF* and *MEK* inhibition) might be preferred to ICI in this patient population. Given the superiority of dual ICI to combination *BRAF* and *MEK* inhibition for extracranial disease, a combination of local therapeutic modalities such as radiotherapy for intracranial disease and ICI to maintain systemic control may be the preferred treatment approach for *BRAF* V600E variant brain metastases.

### Limitations

Our results should be interpreted carefully in the context of key limitations. First, our study was conducted at a single institution, with heterogeneous patient and treatment characteristics; many patients received multiple therapeutic agents, which may have introduced important confounders that could not be controlled for. In addition, the true underlying association between the alterations identified in our cohort (namely, *BRAF* V600E) and the clinical outcomes reported in this study is limited by our retrospective study design and all its inherent limitations, including the presence of residual confounders, selection bias due to missing data, informative censoring, and measurement errors (including potential ambiguity in assessment of radiographic progression subject to clinician interpretation). Our sample size limits broad extrapolation of our results, the patient population treated at a tertiary cancer center undergoing detailed molecular profiling likely differs from the population of all patients with metastatic melanoma, and the snRNA-seq data in particular reflect a high-dimensional data set with only a small number of patient specimens tested. In addition, our targeted sequencing assay is not unbiased and may have missed important additional molecular features such as noncoding variants or UV signature. Futhermore, the lack of sufficient tissue to perform orthogonal antibody-based validation, such as immunohistochemistry, to confirm the observed differences in microenvironment populations prevented broader validation of the CD45-positive observations within the larger brain metastasis cohort. Future work to expand our results to larger, multi-institutional cohorts validated in the prospective setting will be critical to generalize the association between brain metastasis alteration status, cellular landscape, and clinical outcomes.

## Conclusions

This cohort study integrates bulk and single-cell genomic analysis of resected melanoma brain metastases with detailed clinical follow-up to identify *BRAF* V600 alteration status as an important biomarker associated with an altered intracranial tumor microenvironment and decreased response to immunotherapy. Patients with *BRAF* V600E variant melanoma brain metastases may thus benefit from alternative CNS-penetrant systemic regimens.

## Notes

Supplement 1. **eTable 1.** Compiled Variant List From the UCSF500 Assay, a CLIA Certified, Capture Based Targeted DNA Sequencing Assay of 529 Cancer-Associated Genes **eTable 2.** Baseline Clinical and Treatment Characteristics for Patient Cohort by *BRAF* Mutation Status **eTable 3.** Cluster Breakdown by Tumor for Single Nuclear RNA-Sequencing of Melanoma Brain Metastases **eTable 4.** Cluster Marker Gene Lists for Single Nuclear RNA-Sequencing of Melanoma Brain Metastases

Supplement 2. **eFigure 1.** Molecular Analysis From Targeted DNA Sequencing of Melanoma Brain Metastases **eFigure 2.** Clinical Outcomes Based on *BRAF V600E* Status in Patients With Melanoma Brain Metastases **eFigure 3.** Single Nuclear RNA-Sequencing (snRNA-seq) Analysis of Treatment Naïve Melanoma Brain Metastases

Supplement 3.
Data Sharing Statement
