# Supplementary material for: Molecular Features of Resected Melanoma Brain Metastases, Clinical Outcomes, and Responses to Immunotherapy
Source: JAMA Netw Open. 2023 Aug 17;6(8):e2329186. doi: 10.1001/jamanetworkopen.2023.29186 (PMC10436135; doi:10.1001/jamanetworkopen.2023.29186)
Supplement: Supplement 2. — eFigure 1. Molecular Analysis From Targeted DNA Sequencing of Melanoma Brain Metastases eFigure 2. Clinical Outcomes Based on BRAF V600E Status in Patients With Melanoma Brain Metastases eFigure 3. Single Nuclear RNA-Sequencing (snRNA-seq) Analysis of Treatment Naïve Melanoma Brain Metastases [file jamanetwopen-e2329186-s002.pdf]

Supplementary Online Content

Vasudevan HN, Delley C, Chen WC, et al. Molecular features of resected melanoma brain metastases, clinical outcomes, and responses to immunotherapy. *JAMA Netw Open.* 2023;6(8):e2329186. doi:10.1001/jamanetworkopen.2023.29186

eFigure 1. Molecular Analysis From Targeted DNA Sequencing of Melanoma Brain Metastases
eFigure 2. Clinical Outcomes Based on *BRAF V600E* Status in Patients With Melanoma Brain Metastases
eFigure 3. Single Nuclear RNA-Sequencing (snRNA-seq) Analysis of Treatment Naïve Melanoma Brain Metastases

This supplementary material has been provided by the authors to give readers additional information about their work.

© 2023 Vasudevan HN et al. *JAMA Network Open.*

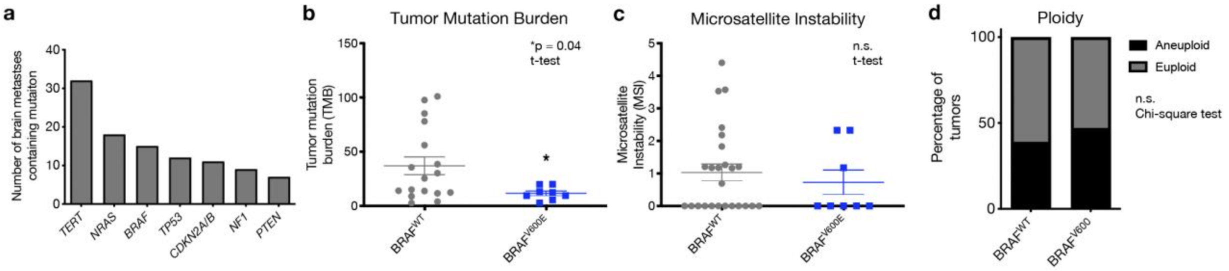

eFigure 1. Molecular Analysis From Targeted DNA Sequencing of Melanoma Brain Metastases**.** a. Recurrent mutations found in melanoma brain metastases include *TERT* alterations (n=32 or 71%), *NRAS* mutation (n=18 or 40%), *BRAF* mutation (n=15 or 33%), *TP53* loss (n=12 or 27%), *CDKN2A/B* loss (n=11 or 24%), *NF1* loss (n=9 or 20%), and *PTEN* loss (n=7 or 16%). b. Tumor mutation burden (TMB) analysis reveals *BRAF* mutant tumors have a significantly lower TMB compared to *BRAF* wildtype tumors (p=0.04, t-test). c. Microsatellite instability analysis (MSI) by *BRAF* status reveals no significant difference (p=0.56, t-test). d. Ploidy estimation based on *BRAF* status reveals no significant difference (p=0.25; Chi-Square test).

© 2023 Vasudevan HN et al. *JAMA Network Open.*

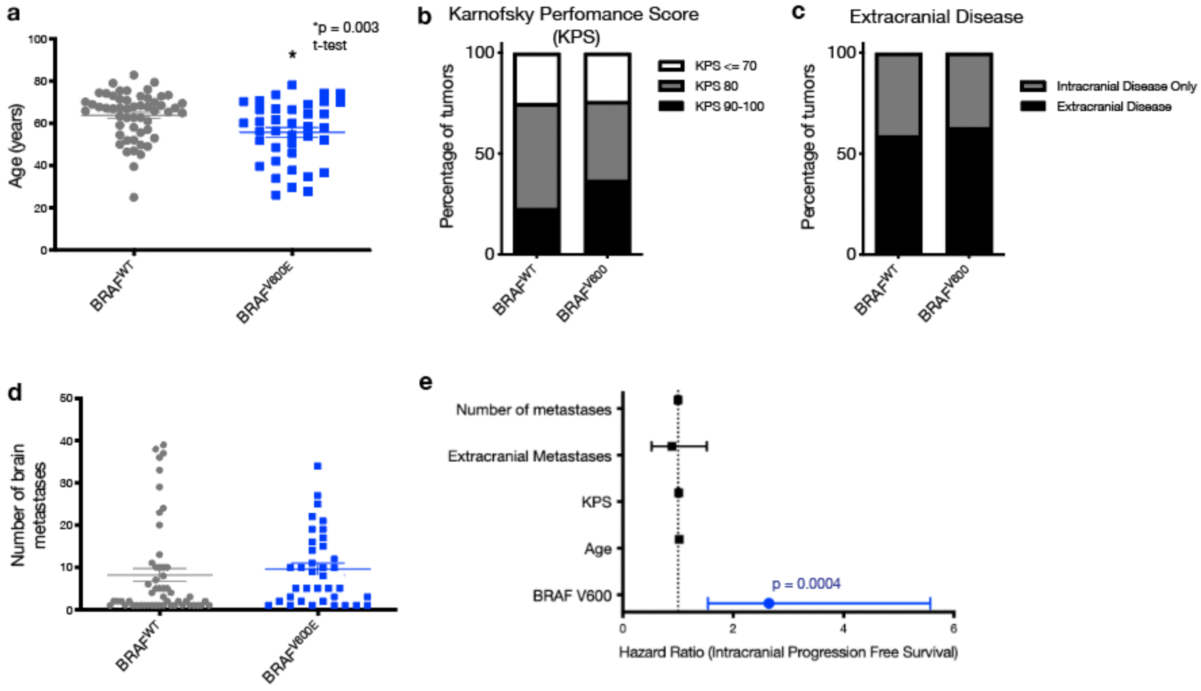

eFigure 2. Clinical Outcomes Based on *BRAF V600E* Status in Patients With Melanoma Brain Metastases. a. Analysis of baseline clinical parameters in *BRAF* mutant versus *BRAF* wildtype melanoma brain metastases reveals patients with *BRAF V600E* mutant melanoma brain metastases are significantly younger (p=0.003, t-test) but no significant differences in b. Karnofsky performance status (KPS) (p=0.07; Chi-square test), c. extracranial disease (p=0.56, Chi-square test), or d. number of brain metastases (p=0.51, t-test). e. Multivariable Cox proportional hazards analysis reveals *BRAF* status is a significant independent factor associated with worse intracranial progression free survival when accounting for age, KPS, or presence of extracranial disease (data plotted as average +/- 95% confidence interval).

© 2023 Vasudevan HN et al. *JAMA Network Open.*

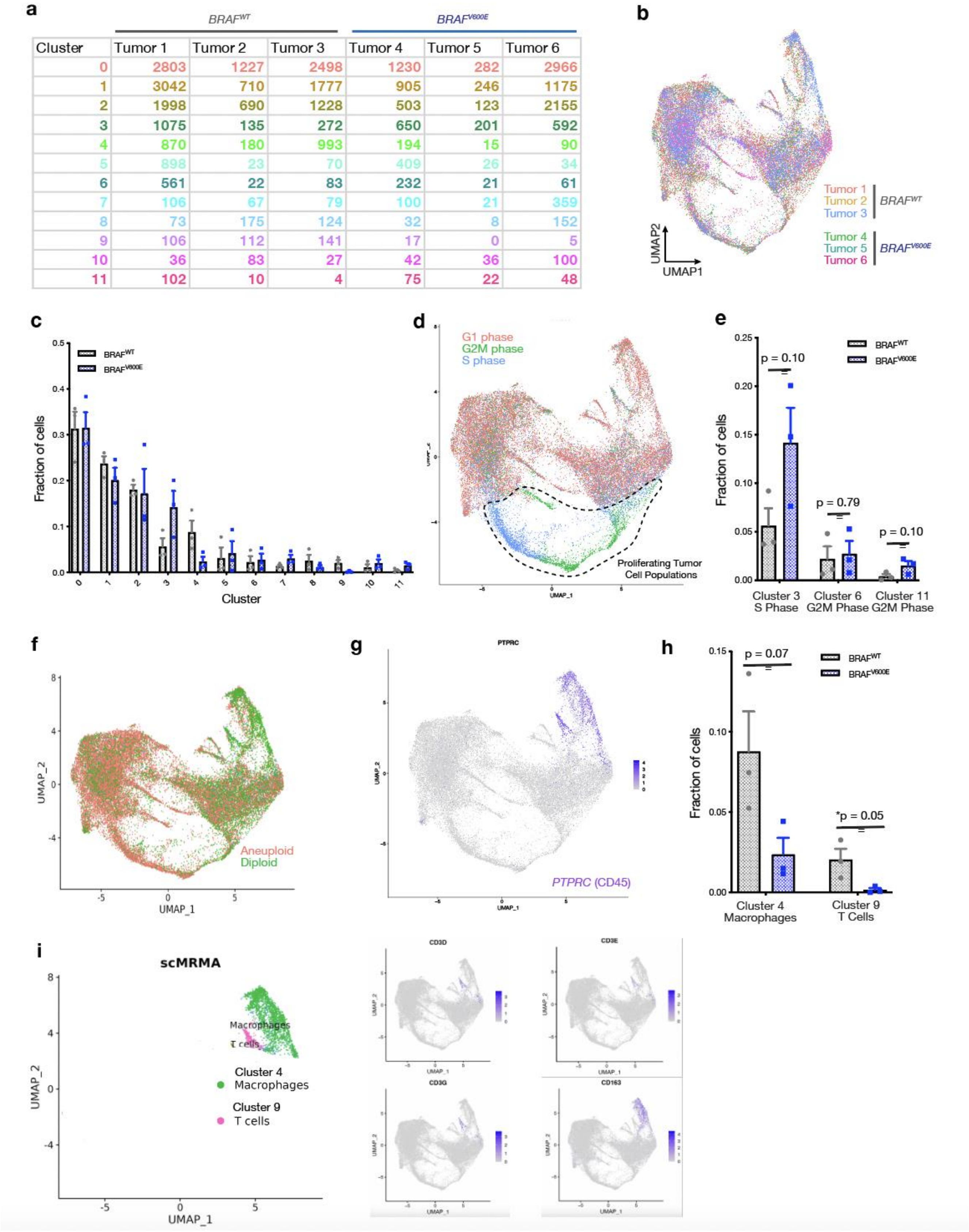

Cluster	Tumor 1	Tumor 2	Tumor 3	Tumor 4	Tumor 5	Tumor 6
0	2803	1227	2498	1230	282	2966
1	3042	710	1777	905	246	1175
2	1998	690	1228	503	123	2155
3	1075	135	272	650	201	592
4	870	180	993	194	15	90
5	898	23	70	409	26	34
6	561	22	83	232	21	61
7	106	67	79	100	21	359
8	73	175	124	32	8	152
9	106	112	141	17	0	5
10	36	83	27	42	36	100
11	102	10	4	75	22	48

© 2023 Vasudevan HN et al. *JAMA Network Open.*

eFigure 3. Single Nuclear RNA-Sequencing (snRNA-seq) Analysis of Treatment Naïve Melanoma Brain Metastases. a. A total of 35,527 single nuclei were isolated and sequenced from n=6 treatment naive *BRAFV600E* mutant (n=3) and *BRAF* wildtype (n=3) surgically resected, frozen brain metastases, leading to the identification of 12 cellular populations. b. Uniform Manifold Approximation and Projection (UMAP) analysis of all 6 samples colored by tumor type. c. Breakdown of cluster composition by *BRAF* mutation status across all 6 brain metastases. d. Cell cycle analysis suggest tumor cell populations in e. *BRAF V600E* mutant samples demonstrate greater proportions of actively dividing cells compared to *BRAF* wildtype samples. f. Single Cell Variational Aneuploidy Analysis (SCEVAN) reveals a common set of normal diploid cells in the top right of UMAP space (green diploid cells). g. Evaluation of *PTPRC* expression (encoding the CD45 protein) coincides with the population of shared, normal diploid cells called by SCEVAN. h. *BRAF V600E* mutant cells trend toward decreased Cluster 4 Macrophages (p=0.16, Mann Whitney test) and Cluster 9 T cells (p=0.10, Mann Whitney test) compared to *BRAF* wildtype cells. i. Automated cell classification and marker gene analysis reveals the CD45+ microenvironment populations correspond to CD3+ T cells and CD163+ macrophages. j. Heatmap showing top 10 cluster marker genes for all 12 clusters.

© 2023 Vasudevan HN et al. *JAMA Network Open.*
