# Supplementary material for: Molecular Features of Resected Melanoma Brain Metastases, Clinical Outcomes, and Responses to Immunotherapy
Source: JAMA Netw Open. 2023 Aug 17;6(8):e2329186. doi: 10.1001/jamanetworkopen.2023.29186 (PMC10436135; doi:10.1001/jamanetworkopen.2023.29186)
Supplement: Supplement 3. — Data Sharing Statement [file jamanetwopen-e2329186-s003.pdf]

# Data Sharing Statement

Vasudevan. Molecular Features of Resected Melanoma Brain Metastases, Clinical Outcomes, and Responses to Immunotherapy. *JAMA Netw Open*. Published August 17, 2023. doi:10.1001/jamanetworkopen.2023.29186

**Data**
**Data available:** No

**Additional Information**
**Explanation for why data not available:** We are happy to provide de-identified information upon request
